# Prevalence and correlates of suspected dementia in older adults receiving primary healthcare in Wuhan, China: A multicenter cross-sectional survey

**DOI:** 10.3389/fpubh.2022.1032118

**Published:** 2022-10-04

**Authors:** Zong-Qin Wang, Lei Fei, Yan-Min Xu, Fang Deng, Bao-Liang Zhong

**Affiliations:** ^1^Department of Psychiatry, Wuhan Mental Health Center, Wuhan, China; ^2^Department of Clinical Psychology, Wuhan Hospital for Psychotherapy, Wuhan, China; ^3^Department of Ultrasound, Renmin Hospital, Hubei University of Medicine, Shiyan, China

**Keywords:** older adults, primary healthcare, dementia, cross-sectional survey, China

## Abstract

**Background:**

Integrating the management of dementia into primary healthcare is a cost-effective way to reduce the burden of dementia but the clinical epidemiology of dementia in primary healthcare settings remains unclear. This study investigated the prevalence and correlates of suspected dementia in Chinese older adults receiving primary healthcare.

**Methods:**

In this multicenter cross-sectional survey, a total of 773 older adults (≥65 years) were consecutively recruited from seven urban and six rural primary care clinics in Wuhan, China, and interviewed with the validated Chinese version of the Brief Community Screening Instrument for Dementia (BCSI-D). Participants with suspected dementia were those who were screened positive on the BCSI-D.

**Results:**

The prevalence of suspected dementia in older primary healthcare adults was 26.8%. Factors significantly associated with suspected dementia were female sex (OR = 1.95, *P* < 0.001), age-group of 75+ (OR = 1.68, *P* = 0.004), poor financial status (OR = 4.79, *P* < 0.001), rural residence (OR = 1.47, *P* = 0.032), no regular physical exercise (OR = 1.74, *P* = 0.002), and stroke and other cerebrovascular diseases (OR = 1.97, *P* = 0.015).

**Conclusions:**

Chinese older adults receiving primary healthcare are at high risk of suspected dementia. Screening and intervention efforts for dementia in primary healthcare settings may be more useful to target older adults who are women, are 75 years and above, have poor economic status, are rural residents, have no exercise habit, and suffer from cerebrovascular diseases.

## Introduction

The Chinese population is rapidly aging with the care and management of older adults with dementia and other cognitive impairments being one of the significant public health concerns in China (1–3). According to the official population statistics, the Chinese elderly population (65 years and above) amounted to 190.6 million in 2020, accounting for 13.5% of the total population in China (4), and, by 2050, this population will continue to grow and will reach 394 million, accounting for 30.2% of the total population (5). Meanwhile, the number of people living with dementia (PLwD) will triple from 16.3 million in 2020 to 49.0 million in 2050 (6). Unfortunately, the healthcare system in China has not been well-prepared to meet the substantial healthcare needs of PLwD (7, 8).

Primary healthcare is usually the first place of contact for older adults with memory problems or other symptoms of cognitive impairment (9). In China, because of the filial piety model of family centered care, 85% of the PLwD live at home and are cared by their family/informal caregivers (7, 10–12). In the context of the shortage of qualified specialists in old age psychiatry and neurology and the wide availability and accessibility of primary healthcare services in communities in China, primary healthcare settings provide a good avenue for the early recognition of older adults with cognitive disorders (13, 14). For example, primary healthcare physicians (PCPs) can be trained to acquire skills to screen for older adults with mild cognitive impairment (MCI) and early dementia. Accordingly, in recent years, there have been increasing calls for integrating dementia management into primary healthcare to improve the early diagnosis of dementia and reduce the caregiver burden of dementia in China (15–17).

To facilitate the planning of the management of cognitive disorders in primary healthcare settings, it is necessary to understand the clinical epidemiology of dementia and other cognitive impairments in Chinese older adults in primary care settings. However, to our knowledge, only two empirical studies have examined the prevalence and associated factors of cognitive impairment in older adults of urban primary healthcare clinics in Shanghai, China (18, 19). The two studies assessed the presence of cognitive impairment by using Mini-Mental State Examination (MMSE) and Montreal Cognitive Assessment (MoCA) and found that the prevalence of suspected dementia (a MMSE score of ≤17 for illiterate participants, 20 for primary school, and 24 for middle school and above) and MCI was 7.8% and 50.1–72.0%, respectively. Factors associated with cognitive impairment included advanced age, diabetes, family history of dementia, hearing loss, and non-participation of social activities. Nevertheless, since the completion of the two patient-based screeners of cognitive impairment, MMSE and MoCA, requires communication and reading ability of the respondents (20), the above two studies excluded older adults who were not able to complete the cognitive tests, possibly resulting in selection bias in their study samples. In addition, since no older adults of rural primary healthcare clinics were recruited in the above two studies, the clinical epidemiology of cognitive impairment in Chinese rural primary healthcare settings remains unknown. Studies using both patient- and informant-based cognitive screeners and recruiting samples of older adults from both urban and rural primary healthcare settings are warranted to address these limitations of prior studies.

In the international literature, there have been several studies investigating suspected dementia in older primary healthcare patients (21–25). Most of these studies were conducted in western countries and the dementia screeners used included MMSE and General Practitioner Assessment of Cognition scale, and the Cambridge Examination for Mental Disorders of the Elderly Cognitive Scale-Revised. However, the reported prevalence of suspected dementia ranged widely, from 5.9 to 56.1%, and only two of them reported a limited number of significant correlates of suspected dementia: female sex, advanced age, a low level of education, and nervous system disease (22, 24). Therefore, knowledge on the clinical epidemiology of suspected dementia among older adults in primary healthcare settings in countries other than China is still very limited.

To fill the above knowledge gaps, this study was set out to investigate the prevalence and correlates of suspected dementia in older adults receiving primary healthcare in both urban and rural areas of Wuhan, the largest city in central China with more than 10 million residents (26).

## Materials and methods

### Participants

The present study was part of a large-scale multicenter cross-sectional survey that examined a range of mental health outcomes in a representative sample of older outpatients receiving primary healthcare in seven urban and six rural primary care clinics in Wuhan, China, between October 2015 and November 2016. Details of the sampling procedures have been published elsewhere (13, 27–29). In brief, Wuhan has 13 districts (seven urban and six rural), so the first stage of the sampling considered the geographic representativeness of the study sample and purposively selected one primary healthcare clinic from each district, which was located in or nearest to the center of the most populous area of the district. In the second stage of the sampling, adults who were at least 65 years old and sought treatment at the 13 selected primary healthcare clinics during the survey period were considered eligible and consecutively invited to participate in this study.

In our pilot study, the prevalence of suspected dementia was 24% in a small sample of 50 older primary healthcare patients. Accordingly, parameters used for the sample size estimation were set as below: the 24% prevalence, a 0.07 confidence interval (CI) width, a two-sided 0.05 type I error rate, and an 80% response rate. By using the formula for sample size estimation for cross-sectional studies, the minimum sample size needed was 749 (30).

The study was approved by the Institutional Review Board of Wuhan Mental Health Center (approval number: WMHC-IRB-S065). All participants provided written informed consent before the interview. Due to the impaired capacity for informed consent of some patients with severe cognitive impairment, their written informed consent was obtained from their family caregivers or other legal guardians.

### Instruments

The survey questionnaire was completed in a face-to-face interview manner. Interviewees included older adults and their caregivers (when present). The interviewers were 13 PCPs who had been trained and were qualified to administer the questionnaire in a standardized way.

The first part of the questionnaire collected sociodemographic, lifestyle, and health-related variables, including sex, age, education, marital status, self-rated financial status (good, fair, poor), main occupation before older adulthood (mental vs. physical labor), residence place (urban vs. rural), living arrangement (alone vs. with others), currently smoking, and regular participation in physical activities.

A checklist was used to assess the presence of chronic medical conditions, including hypertension, diabetes, heart disease, stroke and other cerebrovascular diseases, and chronic obstructive pulmonary disease. Hearing and vision difficulties were operationally defined in this study. During the interview, if the interviewer must speak more loudly than normal to let the elderly know what the interviewer is saying, hearing difficulties was present, while vision difficulties was present if the participant endorsed having difficulties in watching movies or TV shows (27, 31).

The second part of the questionnaire was the Brief Community Screening Instrument for Dementia (BCSI-D), which is an education-fair screening instrument for dementia and suitable for use by non-specialists such as PCPs (20). The BCSI-D has 13 items consisting of two components, a seven-item cognitive test for the participant and a six-item informant interview regarding the participant's performance in everyday living. The Chinese BCSI-D has been proved to be valid for screening for older adults with dementia with the optimal cut-off score of ≤4 for the cognitive test, ≥2 for the informant scale, or ≤4 for the combined algorithm between the cognitive test and the informant scale (32). The sensitivity and specificity of the cognitive test, the informant scale, and the combined algorithm of BCSI-D for screening for dementia in Chinese older adults are 90.7 and 88.6%, 79.1 and 93.3%, and 95.4 and 85.8%, respectively (32). Because the inclusion of informant data additionally improves the screening accuracy of the BCSI-D for dementia (32, 33), our diagnosis of suspected dementia was made primarily based on the combined algorithm score. If the combined algorithm scores were not available, the diagnosis was made based on the cognitive test score alone or the informant scale score alone. In general, a dementia screener for routine use in primary healthcare practice should be brief, easy to administer and score, clinically acceptable to older adults, minimally affected by educational attainment, accurate, and sensitive in particular to mild or early-stage dementia (34). Compared to MMSE and MoCA, BCSI-D is characterized by its good education-fair properties, a low demand of reading and writing abilities, and a short administration time (~6 min) (20). Further, dementia screening with the BCSI-D can be completed with information from either the patient alone or the informant alone (20). Therefore, BCSI-D was selected as the dementia screener of this study. In concordance with earlier studies, participants with suspected dementia were those who were screened positive on the dementia screeners, BCSI-D in this study (35, 36).

### Statistical analysis

Data analyses were conducted with SPSS version 12.0. Prevalence of suspected dementia was calculated. We used Chi-square test to compare rates of suspected dementia between subgroups according to sociodemographic and other characteristics and their corresponding counterparts. A binary logistic regression analysis that entered all statistically significant factors from the Chi-square test as independent variables and suspected dementia as the outcome variable was conducted to identify correlates of suspected dementia. The backward stepwise method was used to select factors. Odds ratios (ORs) with the 95%CIs were used to quantify the associations between factors and suspected dementia. Two-sided *P* < 0.05 was set as the statistical significance level.

## Results

In total, 791 older adults receiving primary healthcare were invited to join the study and 773 completed the survey questionnaire. Among the 773 completers of the BCSI-D, 317 (41.0%) completed the cognitive test only, one (0.1%) completed the informant scale only, and 455 (58.9%) completed both the cognitive test and the informant scale. The mean age of the study sample was 72.9 years (standard deviation = 6.1, range = 65–97) and 414 (53.6%) were women. Table 1 displays the characteristics of the survey sample.

**Table 1:** Characteristics of the sample of older adults receiving primary healthcare and prevalence rates of suspected dementia by factor.

**Characteristics**		***n***	**No. of older adults with suspected dementia**	**Prevalence (%)**	**χ^2^**	***P*** **-value**
Sex	Male	359	75	20.9	11.849	0.001
	Female	414	132	31.9		
Age (years)	65–74	495	116	23.4	7.852	0.005
	75+	278	91	32.7		
Education	Illiterate	183	76	41.5	30.241	< 0.001
	Primary school	221	64	29.0		
	Middle school and above	369	67	18.2		
Marital status	Married	544	140	25.7	1.02	0.313
	Others^*^	229	67	29.3		
Self-rated financial status	Good	136	23	16.9	33.607	< 0.001
	Fair	546	138	25.3		
	Poor	91	46	50.5		
Main occupation before older adulthood	Mental labor	221	36	16.3	17.366	< 0.001
	Manual labor	552	171	31.0		
Residence place	Urban	411	93	22.6	7.713	0.005
	Rural	362	114	31.5		
Living alone	No	687	186	27.1	0.275	0.600
	Yes	86	21	24.4		
Current smoking	No	652	185	28.4	5.407	0.020
	Yes	121	22	18.2		
Regular physical excise	No	439	88	20.0	23.492	< 0.001
	Yes	334	119	35.6		
Hypertension	No	401	107	26.7	0.004	0.950
	Yes	372	100	26.9		
Diabetes	No	658	173	26.3	0.535	0.465
	Yes	115	34	29.6		
Heart disease	No	693	186	26.8	0.013	0.910
	Yes	80	21	26.3		
Stroke and other cerebrovascular diseases	No	706	180	25.5	6.838	0.009
	Yes	67	27	40.3		
Chronic obstructive pulmonary disease	No	728	193	26.5	0.457	0.499
	Yes	45	14	31.1		
Hearing difficulties	No	743	203	27.3	2.878	0.090
	Yes	30	4	13.3		
Vision difficulties	No	696	195	28.0	5.466	0.019
	Yes	77	12	15.6		

* “Others” included never married, separated, divorced, widowed, cohabitating, and remarried.

Altogether, 207 older adults were screened positive by the BCSI-D and the corresponding prevalence of suspected dementia was 26.8%. Women had significantly higher prevalence of suspected dementia than men (31.9 vs. 20.9%, *P* = 0.001) and rural older adults had significantly higher prevalence of suspected dementia than urban older adults (31.5 vs. 22.6%, *P* = 0.005). In addition, significantly higher prevalence of suspected dementia was observed in older adults aged 75 years old and above (vs. 65–74 years), illiterate older adults and those with an educational attainment of primary school (vs. middle school and above), those who rated their financial status as “poor” (vs. good), those who engaged in physical labor before older adulthood (vs. mental labor), those who were not currently smoking, those who regularly participated in physical activities, those who suffered from stroke and other cerebrovascular diseases, and those who had vision difficulties (Table 1).

Factors significantly associated with suspected dementia were female sex (OR = 1.95, *P* < 0.001), age-group of 75+ (OR = 1.68, *P* = 0.004), poor financial status (OR = 4.79, *P* < 0.001), rural residence (OR = 1.47, *P* = 0.032), no regular physical exercise (OR = 1.74, *P* = 0.002), and stroke and other cerebrovascular diseases (OR = 1.97, *P* = 0.015) (Table 2).

**Table 2:** Correlates of suspected dementia in older adults receiving primary healthcare.

**Factor**	**Risk level**	**Reference level**	**Co-efficient**	**Standard error**	**χ^2^**	***P*** **-value**	**OR (95%CI)**
Sex	Female	Male	0.668	0.177	14.248	<0.001	1.95 (1.38, 2.76)
Age (years)	75+	64–74	0.517	0.179	8.326	0.004	1.68 (1.18, 2.38)
Self-rated financial status	Poor	Good	1.566	0.324	23.334	<0.001	4.79 (2.54, 9.04)
Residence place	Rural	Urban	0.385	0.179	4.601	0.032	1.47 (1.03, 2.09)
Regular physical exercise	No	Yes	0.552	0.178	9.574	0.002	1.74 (1.22, 2.46)
Stroke and other cerebrovascular diseases	Yes	No	0.676	0.279	5.875	0.015	1.97 (1.14, 3.40)

## Discussion

The World Health Organization advocates the integration of dementia management into primary healthcare to provide sustainable care to PLwD in its member states, in particular in low- and middle-income countries (LMICs) (37), but knowledge on the prevalence and clinical characteristics of dementia in primary healthcare settings remains very limited in China and other LMICs. To the best of our knowledge, the present study is the first study examining the clinical epidemiology of suspected dementia in a representative sample of older adults in both urban and rural primary healthcare settings in China. The main findings are the 26.8% prevalence of suspected dementia in older adults and six significant correlates of suspected dementia in this patient population: female sex, age-group of 75+, poor economic status, rural residence, no exercise habit, and cerebrovascular diseases.

In epidemiological surveys of community-dwelling Chinese older adults, the prevalence of suspected dementia, as defined by the cut-off scores of MMSE and BCSI-D, was 7.8–18.7% (19, 38, 39). In comparison to these estimates, the prevalence of suspected dementia in older primary healthcare patients is higher than that in community-dwelling older adults. In this study, up to 57. 8% of the participants had at least one of the four chronic medical conditions. In prior reports of the same study sample of participants, 20.3 and 26.2% had major depression and felt lonely at least sometimes, respectively (28, 29). Because chronic medical conditions, major depression, and loneliness can increase the risk of dementia (40–42), the elevated risk of suspected dementia in older adults receiving primary healthcare is expected.

Our findings on correlates of suspected dementia are largely consistent with earlier studies on risk factors of dementia (40, 41, 43, 44), confirming the multifactorial etiology of dementia in older primary healthcare patients. For example, women are more likely to develop Alzheimer's disease and advanced age is the most prominent risk factor for dementia (45). Cerebrovascular diseases such as stroke can interrupt the blood supply to some areas of the brain, which in turn results in the death of brain cells and causes memory loss and other symptoms of dementia (46). The significantly higher risk of suspected dementia in older primary healthcare patients with poor financial status confirms the elevated risk of dementia in older adults with a low socioeconomic status (47). The significant association between rural residence and suspected dementia may be ascribed to the urban-rural health disparities such as the poor health literacy in rural older adults and the inadequate provision of health services in rural areas (48, 49).

The significant associations between suspected dementia and two modifiable factors, no regular participation in physical activities and cerebrovascular diseases, are interesting, suggesting the clinical needs of lifestyle interventions and effective management of cerebrovascular diseases in preventing or delaying the progression of dementia in primary healthcare settings.

This study has some limitations. First, the diagnosis of dementia based on diagnostic criteria such as DSM-V is more clinically relevant, but we only assessed the presence of suspected dementia based on BCSI-D in this study. Second, this is a cross-sectional study only, so our findings on correlates of suspected dementia only indicated correlation relationships. Prospective studies are needed further examine the causal relationships between identified factors and suspected dementia. For example, poor economic status could increase the risk of dementia while dementia is also likely to cause economic difficulties. Third, we did not assess the recognition and treatment rates of patients with suspected dementia, which are also important for the planning of old age mental health services at primary healthcare settings. Fourth, our sample of older adults receiving primary healthcare was recruited from a large municipality in central China, older primary healthcare patients of other cities were not included, potentially limiting the generalizability of our findings.

Given the high prevalence of suspected dementia in older primary healthcare patients in China and the high disease burden associated with dementia, it is necessary to train Chinese PCPs to improve their capacity to recognize and help manage dementia. Importantly, to make dementia screening and management as a routine services of primary healthcare clinics, health policy-makers should consider to integrate the management of dementia into China's nationwide Basic Public Health Services Package (50). Based on the findings on correlates of suspected dementia, screening and intervention efforts for dementia in primary healthcare settings may be more useful to target older adults who are women, aged 75 years and above, have poor economic status, are rural residents, have no exercise habit, and suffer from cerebrovascular diseases.

## Data availability statement

The original contributions presented in the study are included in the article/supplementary material, further inquiries can be directed to the corresponding author.

## Ethics statement

The studies involving human participants were reviewed and approved by the Institutional Review Board of Wuhan Mental Health Center. The patients/participants provided their written informed consent to participate in this study.

## Author contributions

Z-QW: acquisition and analysis of data for the study, drafting the paper, and interpretation of data for the study. LF and FD: design and acquisition of data for the study. Y-MX and B-LZ: drafting the paper, revising the paper for important intellectual content, and interpretation of data for the study. All authors contributed to the article and approved the submitted version.

## Funding

This work was supported by National Natural Science Foundation of China (grant number: 71774060), 2015 Irma and Paul Milstein Program for Senior Health Awards from the Milstein Medical Asian American Partnership Foundation, the Young Top Talent Program in Public Health from Health Commission of Hubei Province (PI: B-LZ), and Wuhan Health and Family Planning Commission (grant numbers: WX17Q30; WG16A02; WG14C24).

## Conflict of interest

The authors declare that the research was conducted in the absence of any commercial or financial relationships that could be construed as a potential conflict of interest.

## Publisher's note

All claims expressed in this article are solely those of the authors and do not necessarily represent those of their affiliated organizations, or those of the publisher, the editors and the reviewers. Any product that may be evaluated in this article, or claim that may be made by its manufacturer, is not guaranteed or endorsed by the publisher.
